# Intestinal Parasitic Infections Among Pediatric Patients in a Metropolitan City of Bangladesh With Emphasis on Cryptosporidiosis

**DOI:** 10.7759/cureus.26927

**Published:** 2022-07-16

**Authors:** Nusrat Jahan Nipa, Nasima Aktar, Hasina M Hira, Farhana Akter, Dilshad Jahan, Salequl Islam, Ayukafangha Etando, Adnan Abdullah, Kona Chowdhury, Rahnuma Ahmad, Ahsanul Haq, Mainul Haque

**Affiliations:** 1 Microbiology, Rangamati Medical College, Rangamati, BGD; 2 Microbiology, Chittagong Medical College, Chattogram, BGD; 3 Community Medicine, Chittagong Medical College, Chattogram, BGD; 4 Endocrinology and Diabetes, Chittagong Medical College, Chattogram, BGD; 5 Hematology, Asgar Ali Hospital, Dhaka, BGD; 6 Microbiology, Jahangirnagar University, Savar, BGD; 7 Medical Laboratory Sciences, Faculty of Health Sciences, Eswatini Medical Christian University, Mbabane, SWZ; 8 Occupational Medicine, Faculty of Medicine and Defence Health, Universiti Pertahanan Nasional Malaysia (National Defence University of Malaysia), Kuala Lumpur, MYS; 9 Pediatrics, Gonoshasthaya Samaj Vittik Medical College, Savar, BGD; 10 Physiology, Medical College for Women and Hospital, Dhaka, BGD; 11 Statistics, Gonoshasthaya – RNA Biotech Limited, Savar, BGD; 12 Pharmacology and Therapeutics, Universiti Pertahanan Nasional Malaysia (National Defence University of Malaysia), Kuala Lumpur, MYS

**Keywords:** cryptosporidium infection, chattogram, cryptosporidiosis, pediatric patients, diseases, contagions, intestinal parasitic

## Abstract

Introduction

Gastrointestinal parasitic infections are one of the global health concerns in developing countries like Bangladesh. Among them, *Cryptosporidium* spp. plays an essential role in causing diarrhea, malnutrition, and poor cognitive function, especially in children. This study was conducted to identify the frequency of *Cryptosporidium* cases and other parasitic agents.

Methods

A cross-sectional observational study was conducted among 219 hospitalized children with diarrhea. The conventional microscopic technique was applied for parasitic detection. Particular staining (modified Ziehl-Neelsen) procedure was performed to identify oocysts of *Cryptosporidium *spp*. *A polymerase chain reaction (PCR) was performed to determine the *SSU rRNA* and *gp60 *gene of *Cryptosporidium. *

Results

Cysts of *Giardia duodenalis* (2.3%), ova of *Ascaris lumbricoides *(1.4%,), *Trichuris trichiura *(0.5%), and both *A. lumbricoides* and *T. trichiura* (0.9%) were identified in samples through wet mount preparation. The distribution of *Cryptosporidium* spp. as detected by the staining method and nested PCR was 1.4% and 4.1%, respectively.

Conclusion

Factors independently associated with *Cryptosporidium *infection are unsafe water, lack of regular hand washing, and insufficiency of exclusive breastfeeding. This study reports, presumably for the first time, the detection of *Cryptosporidium* oocysts in Chattogram metropolitan city of Bangladesh.

## Introduction

Diarrhea is the passage of three or more liquid or loose stools per day or if the individual experiences more frequent passage than usual [1-3]. It is categorized clinically as i) acute watery diarrhea, which persists for several hours or days [4], ii) persistent diarrhea that lasts for 14 days or longer [5], and finally, iii) dysentery, blood, and mucous found in diarrheal stool [6,7]. Previously, diarrhea was one of the deadliest diseases [8,9] and remains the second dominant cause of child mortality worldwide [10,11]. Almost 1.7 billion cases of childhood diarrheal diseases are diagnosed each year globally, resulting in the annual death of approximately 525,000 children under five, around 63% of the reported global diarrhea burden [12]. Consequently, acute diarrheal disease remains the top cause of morbidity and mortality among the pediatric population after a respiratory illness, especially in low-middle-income countries, and creates a serious public health issue [13,14]. It is liable for one in eight deaths among children less than five years in Africa, Asia, and South America per annum, or roughly 499,000 children are incriminated of diarrheal disease [13]. The vast majority occurs in Sub-Saharan Africa, reflecting this region's highest child death rate [14-16].

A single episode of moderate-to-severe diarrhea has significant repercussions on mortality and linear growth among survivors, facilitating the hazards of growth retardation, ill health, and cognitive impairment in the pediatric community [17-19]. Diarrheal diseases are a significant public health problem that affects children in developing countries where insufficient sanitation, hygiene, and portable water supply are the critical factors [13,20-25]. In Bangladesh, one-third of the child death burden is due to diarrhea, a life-threatening disease [26-28]. It was observed that a rural child suffers from 4.6 episodes of diarrhea and about 230,000 children die among them per year [26]. Therefore, it is essential to identify the etiology with the proper diagnostic procedures, followed by disease management interventions are then possible.

Etiological agents of diarrhea

The rotavirus is the most prevalent among numerous viral, bacterial, and parasitic agents causing pediatric diarrhea [29,30]. Some bacterial enteropathogens are also responsible for generating the same, such as *Shigella*, nontyphoidal *Salmonella* (NTS), Diarrhoeagenic *Escherichia coli* (DEC), *Vibrio cholerae*, *Campylobacter*, and *Yersinia* spp. [23,31,32]. Although enteric viruses and bacteria remain the predominant etiological agents [33-35], intestinal protozoal parasites also add related helminths that are also significantly related to diarrheal disease in children, including *Cryptosporidium* spp., *Giardia duodenalis,* *Entamoeba histolytica*, *Blastocystis hominis*, *Dientamoeba fragilis, Ascaris lumbricoides, Trichuris trichiura*, *Ancylostoma duodenale*, and *Necator americanus* [36-38].

*Cryptosporidium* as a protozoan diarrheal agent

An intracellular apicomplexan protozoan parasite belonging to the genus *Cryptosporidium* is second only to rotavirus as the leading cause of moderate-to-severe diarrhea [39-41]. The organism attracts significance as one of the most prominent causes of diarrhea and diarrhea-causing death in young children, especially among infants and immunodeficient individuals in developing countries worldwide [40,42,43]. As the annual detection rate of *Cryptosporidium*-ascribable cases is about 2.9-4.7 million in children under two years, the sub-Saharan African and South Asian territories face tremendous challenges in combating this infection [44].

Various species of this parasite have been identified and distinguished regarding host range and public health concerns [43]. Thus,* Cryptosporidium*, the ubiquitous coccidian parasite, has over 40 established species, and of these, 20 species and subtypes account for the vast majority of human gastrointestinal infections worldwide. Additionally, *C. hominis *and *C. parvum *are the leading culprits causing cryptosporidiosis globally [45, 46]. Transmission occurs with fecal-oral transmission, either by zoonotic or anthroponotic transmission. *Cryptosporidium* spp. has a low infective threshold with a robust oocyst that survives adequately in moist, ambient environments and is resistant to many commonly available disinfectants such as chlorine [18,47].

Effects of Cryptosporidiosis

Diarrhea associated with cryptosporidiosis has been linked to three fundamental mechanisms [40,48,49]: i) osmotic diarrhea caused by malabsorption [50,51]; ii) parasite-induced production of inflammatory products and host neurohumoral secretagogues [48,49], and iii) secretory diarrhea caused by a parasite enterotoxin [52-54]. Different absorptive and secretory characteristics exist in different parts of the gastrointestinal system [55]. In immunocompetent persons, the small intestine, principally the ileum, is the primary location of *Cryptosporidium* infection. However, in AIDS patients, the gastrointestinal parasite distribution is more complex and extensive [56,57].

The infection ranges from asymptomatic, self-limiting diarrhea to chronic [58-60]. The disease's intensity depends on the individual host's age, nutrition, and immune status [18]. The disease disrupts the intestinal epithelium, impairs the absorptive and barrier function of the small intestine, and initiates prolonged (7-14 days) and persistent (≥14 days) diarrhea [18,61]. Though cryptosporidiosis is a self-limiting disease in immunocompetent individuals, the aftermath of this illness has far-reaching threatening hazards beyond diarrheal consequences. It also interferes with nutrient absorption, resulting in chronic malnutrition, poor growth, and premature mortality, especially in developing countries [37,62]. Despite enlisting *Cryptosporidium* in its "Neglected Disease Initiative 2004" by WHO, it's one of the significant causes of diarrhea in children [63]. Several factors are related to this infection, such as host, environmental, and parasite species. Long-term contact with domestic animals, overcrowded places, poverty, poor sanitation, contaminated water sources, the immune status of the individual, and malnourishment in children also play a crucial role in this infection [40]. Recent studies highlight a reduction in overall diarrheal episodes in Bangladesh by improving water sources and sanitation behavior. Still, cryptosporidiosis and growth faltering, a recognized upshot of this infection, haven't decreased [64,65].

Studies on Cryptosporidium

Epidemiologic studies have demonstrated that *Cryptosporidium* is more prevalent in developing countries than in developed ones [25,40,42,66]. The organism has been reported as a substantial burden causing acute diarrhea [67]. A meta-analysis study on consequences of childhood diarrhea caused by this protozoan infection showed that, in 2016, it was the fifth most significant diarrheal etiological pathogen globally in children younger than five years, and acute infection attributed to more than 48,000 deaths and more than 4·2 million disability-adjusted life-years lost [18]. The significance of this intestinal parasite can easily be realized by the current Global Enteric Multicentric Study (GEMS) done on children from seven Asian and African countries, where 9,439 were moderate-to-severe diarrheal cases and 13,129 control subjects, unveiling four exclusive moderate-to-severe diarrhea-causing agents namely Rotavirus, *Cryptosporidium*, enterotoxigenic *E. coli*, and *Shigella* [68].

In a longitudinal cohort study on 392 Bangladeshi slum-dwelling children (in the first two years of life) performed from 2008 to 2014, *Cryptosporidium* infection was widespread (77%). The study also highlights a close correlation between poverty and stunted growth during the first two years of life [40]. A survey of over 423 fecal samples from 185 children (up to five years) in an urban slum area of Bangladesh was done. *Cryptosporidium* oocyst was detected in 9.2% of cases, where the infection was highest among children aged less than two. Moreover, that study also observed the infection decreases with age [69]. In a prospective study, fecal samples from children under 16 years attending an outpatient clinic in Cambodia were examined for *Cryptosporidium*, where these protozoan oocysts were detected in 2.2-7.7% of cases [61]. In Tehran, a study was done in which stool samples from children below 12 years with diarrhea were collected, and 1.1% of these cases were found positive for this infection [66]. Another Iranian study reported that *Cryptosporidium* oocysts were detected in 3.8-8% of pediatric and immunocompromised patients [68].

Diagnostic modalities

The diagnostic procedure comprises the microscopic examination of the fecal sample by wet mount and staining by modified Ziehl-Neelsen (mZN) staining [68] or auramine-phenol staining technique [70]. Direct and indirect immunofluorescence assays are expensive, but oocysts are readily identified [48,71]. An immunological method (enzyme immunoassay (EIA), enzyme-linked immunoassay (ELISA), and immunochromatographic technique (ICT)) provides good sensitivity over microscopy but has drawbacks, including false-positive results, and is unavailable in developing countries due to cost ineffectiveness [72-74]. Though commonly used and cost-effective, the microscopic procedure is labor-incentive and time-consuming, and the process exclusively relies on an individual's skill and experience [57,75]. Moreover, molecular methods are extensively used for genotyping and molecular epidemiological studies because of their higher sensitivity (detection ranges from 1-106 oocysts) over the traditional microscopic and immunological procedure [45,76,77].

Various genes of *Cryptosporidium* are documented targeting its species [78], such as small subunit rRNA (SSU rRNA), *Cryptosporidium* outer wall protein (COWP), 70-kDa heat shock protein (HSP 70), thrombospondin-related adhesive protein (TRAP-C2), dihydrofolate reductase (DHFR), and actin genes [45,79-81]. *SSU rRNA* and *gp60* are the most common genetic markers for *Cryptosporidium* species identification and subtype determination [82-84]. The *SSU rRNA* gene is considered extensively used in genotypic differentiation between infections belonging to both humans and animals. In addition, the 60-kDa glycoprotein (gp60) gene possesses highly variable regions that permit many intraspecies sequence heterogeneity. These sites are crucial to determining many *C. parvum* and *C. hominis *subspecies [45,57]. The majority of the pathogenic strains are detected by nested assay, which is also chosen for identifying the negligible amount of oocyst (<100) in the specimen [57]. As the parasite has a long-term detrimental effect on childhood growth, nutrition, and cognitive function with no appropriate drug or vaccine strategy, a study on this parasite carries much importance. To date, no such research was previously done in Chattogram city. Hence, this study was designed to detect this pathogen and other parasites as a parasitic etiological cause of diarrhea in pediatric patients in this metropolitan city.

In the current study, microscopy by wet mount preparation, concentration technique (formalin-ether sedimentation technique), and staining procedure (mZN) were applied to all samples. Subsequently, nested PCR was performed targeting *SSU rRNA* and the *gp60* gene. The study would help the clinician diagnose the case properly by exploring the actual etiology of protozoa-related diarrheal illness and indirectly minimizing the empirical use of antibiotics to treat parasitic diarrhea, thus reducing the multi-drug resistant problem in Bangladesh. 

Objectives of the study

The objectives of the study include i) identification of intestinal parasites microscopically by wet mount preparation, ii) identification of *Cryptosporidium* microscopically by mZN staining technique, iii) detection of the presence of *Cryptosporidium* spp. by nested PCR, and iv) comparison of the result of microscopy with that of PCR.

This paper was previously posted to the Preprints preprint server on March 7, 2022 (https://www.preprints.org/manuscript/202203.0108/v1).

## Materials and methods

Study details

This was a cross-sectional observational study conducted in the Department of Microbiology, Chittagong Medical College, Chattogram, Bangladesh, and Department of Pathology and Parasitology, Chittagong Veterinary and Animal Sciences University (CVASU), Chattogram, Bangladesh, from July 2019 to June 2020. The study population included indoor and outdoor pediatric diarrheal patients of Chittagong Medical College Hospital and Chattogram Maa-O-Shishu Hospital Medical College, Chattogram, Bangladesh.

The study received ethical approval from the Institutional Review Board of Chittagong Medical College Hospital (CMCH), Chattogram, Bangladesh (Approval number CMC/PG/2019/592, dated November 3, 2019). The research method was strictly aligned with the Declaration of Helsinki. Participation and reporting were hinged on consent forms signed by patients/guardians of the pediatric diarrheic patients (children up to 18 years) before administering the questionnaire. The respondents were informed correctly using the participant's information sheet about their rights and all the relevant aspects of the study, including its aim and interview procedure. No patient was older than 18 years.

Sample size calculation and technique

n= z2pq/d2==123.2. The n is the sample size. z is the confidence interval (95%) z=1.96. p is the pre-estimated prevalence of 9.2% obtained from a study performed by Ahmed et al. conducted in Dhaka, Bangladesh [85]. d is a marginal error (5%). n = (1.96)2 x0.09 2x0.908/ (0.05) 2=123.17. This means a minimum of 123 participants need to participate in this study.The sampling technique w*as *a nonprobability, purposive type of sampling. 

Eligibility criteria

**Figure 1:**
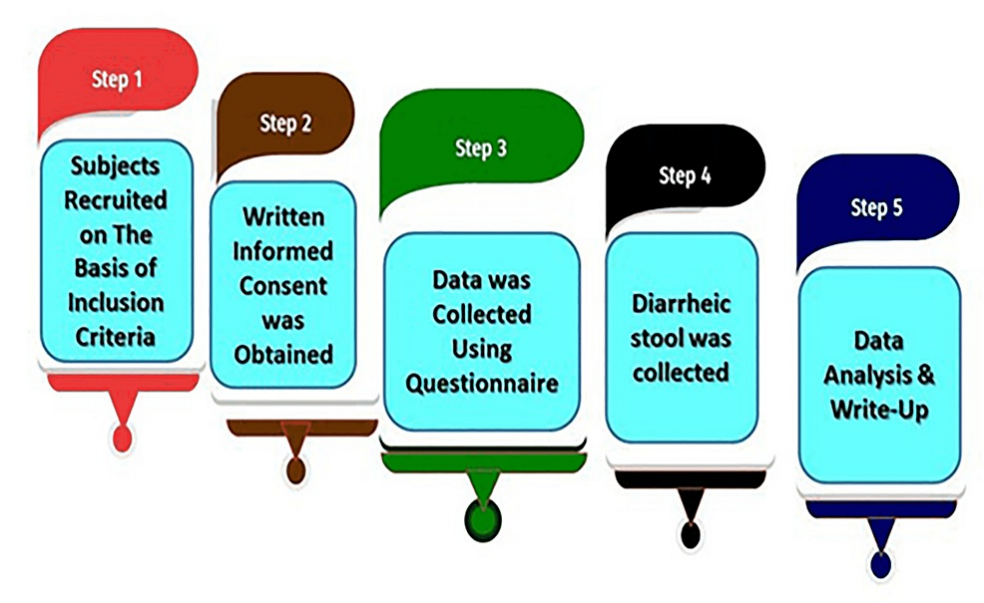
Flowchart depicting the macroscopic study plan

This study was conducted on both inpatients and outpatients. Bangladeshi pediatric patients (up to 18 years) suffering from acute watery and persistent diarrhea (≥14 days) irrespective of their social status [27,86,87], who agreed or whose guardians gave consent to enroll their children after the complete explanation of the study objectives were included.Pediatric patients having bloody diarrhea (with blood and mucous) and above the age of 18 years were excluded. Moreover, patients or guardians who were unwilling to sign the assent form were excluded. The macroscopic study plan is illustrated in Figure 1.

Preparation of questionnaire

A questionnaire was prepared and modified from the study conducted by Tombang et al. in Cameroon [88]. The questionnaire was adjusted using the study's eligibility criteria and Bangladeshi cultural aspects. In the first section of the questionnaire, particulars of the patients were included. This was followed by socio-demographic history, habitual elements, and clinical features.

Sample collection

After taking consent, the patient's history details, including demographic information and clinical findings, were recorded in a predesigned case record form. Then stool sample was collected in a clean, leak-proof, wide-mouth container appropriately labeled with the patient's name, age, time of collection, and identification number. It was then transported to the Department of Microbiology, and some portions of each sample were transferred to the Eppendorf tube and refrigerated at -80ºC to perform the molecular method [89]. The remaining sample was transferred into two containers. One container contained a 10% formalin preserved fecal sample for direct wet mount preparation, and the other retained an unpreserved fecal sample. This was followed by the concentration procedure which consisted of the following step: i) By using a stick, an estimated 1gm of feces was emulisified in about 4 ml of 10% formol water contained in a tube, ii) A further 3-4 ml of 10% v/v formol water was added, the tube was capped, and mixed well by shaking, iii) The emulsified feces were sieved collecting the sieved suspension in a beaker, iv) the solution was transferred to a centrifuge tube and 3-4 ml of ethyl acetate was added, v) The tube was then vortexed for 15 minutes, vi) It was centrifuged immediately at low speed 1000 rpm for one minute, vii) A pipette was used to remove the entire column of fluid below the fecal debris and ether and this was transferred to another centrifuge tube, viii) Formol water was added to make the volume up to 10 to 15 ml and centrifuged at 3000 rpm for 5-10 minutes, ix) The supernatant was removed and the bottom of the tube was tapped to resuspend and mix the sediment [90-92] was followed with this unpreserved sample before the staining procedure. The sediment was now ready to make a smear on the slide for microscopic examination.

Laboratory procedure

**Figure 2:**
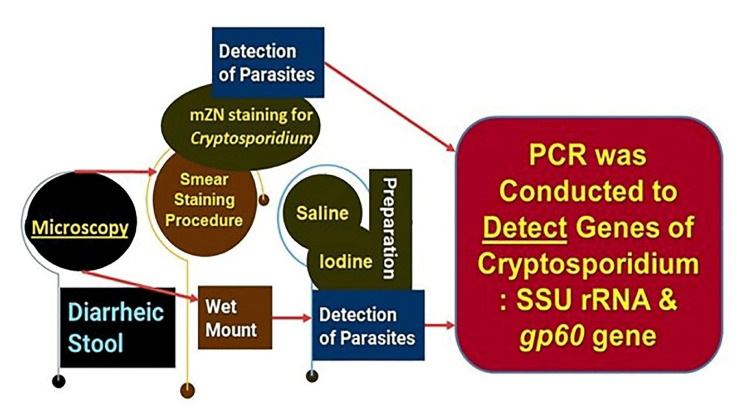
The steps of laboratory methods employed PCR: polymerase chain reaction

**Figure 3:**
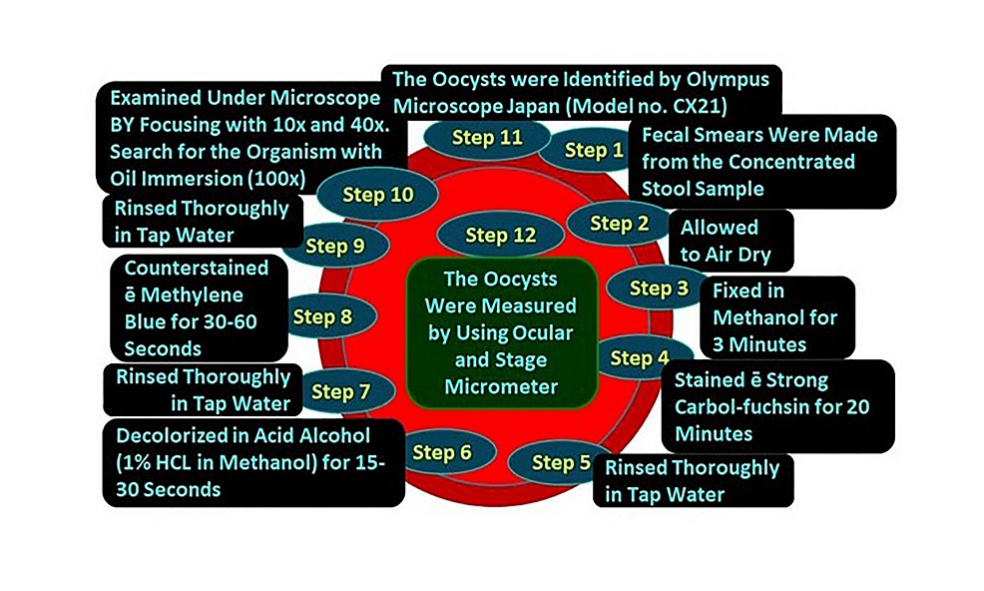
Steps of modified Ziehl-Neelsen stain HCl: hydrochloric acid

All concentrated samples were subjected to wet mount preparation by both saline and iodine preparation (saline wet mounts and iodine wet mount were arranged by discreetly blending a small volume of stool sample with a drop of physiological saline, methylene blue dye, and Lugol's iodine (diluted in 1: 5 distilled water), respectively, on a glass slide and employing a coverslip over the smear) [93,94] and staining by mZN (Figure 2 and Figure 3). A fecal smear was made from a concentrated stool sample on a clean, grease-free glass slide. After fixation, mZN staining was performed and the slide was examined under a microscope using oil immersion. *Cryptosporidium* oocysts were identified as red, small (4-6 µm), characteristically round or slightly ovoid, and acid-fast oocysts against a blue background [95].

Procedure of PCR

Process of DNA Extraction

DNA extraction was performed according to the manufacturer's instruction with the Invitrogen PureLink Microbiome DNA Purification kit (Thermo Fisher Scientific, Waltham, Massachusetts, United States) and the procedure was performed at room temperature (20-25°C). The purified DNA in the tube was preserved at -20°C for further use. Primers used for *SSU rRNA *and* gp60Gene* [96,97] are given in Table 1. The first pair of primers (Table 1) were used as first-round PCR to amplify the 830 bp sequence of the *SSU rRNA* gene, and the second pair of primers were used for second-round PCR to amplify a 240 bp sequence. For the *gp60* gene, first set primers (Table 1) were used as first-round PCR to amplify the 412 bp sequence, and the next pair of primers were used for second-round PCR to amplify the 350 bp sequence of the *gp60* gene.

**Table 1:** Primers used for SSU rRNA and gp60 genes of Cryptosporidium species

		Primer name	Primer sequence (5’-3')	Size
*SSU rRNA* gene	First Set Primer	XF2	F-GGAAGGGTTGTATTTATTAGATAAAG	830 bp
		XR2	R-AAGGAGTAAGGAACAACCTCCA	
	Second Set Primer	PSSUf	F-AAAGCTCGTAGTTGGATTTCTGTT	240 bp
		PSSUr	R-ACCTCTGACTGTTAAATACRAATGC	
*gp60* gene	First Set Primer	gp15-ATG	F-ATGAGATTGTCGCCTCATTATC	412 bp
		gp15-STOP	R-TTACAACACGAATAAGGCTGC	
	Second Set Primer	gp15-15A	F-GCCGTTCCACTCAGAGGAAC	350 bp
		gp15-15E	R-CCACATTACAAATGAAGTGCCGC	

Preparation of Reaction Mixture

Sterile micro-centrifuge tubes (1.5 ml) were taken and labeled with the date and identification number. Primer tubes were centrifuged for a few seconds. Then it was vortexed for 15 seconds and diluted with nuclease-free water to make a 1:10 dilution. For each sample, a total of 20 µl of the mixture was prepared by mixing 10 µl of master mix (mixture of dNTP, Taq Polymerase, MgCl2, and PCR buffer), 1 µl forward primer, 1 µl of reverse primer, 2 µl DNA template, and 6 µl of nuclease-free water.

*Cyclic Condition used for Nested PCR *[98]

*SSU rRNA* Gene of *Cryptosporidium* Species: In the first round PCR*, *initial denaturation at 94°C for five minutes was followed by 30 cycles of denaturation at 94°C for 45 seconds, primer annealing at 45^0^C for two minutes, extension at 72°C for 1.5 minutes, and a final extension at 72°C for 10 minutes. In the second round PCR, initial denaturation at 94°C for five minutes was followed by 35 cycles of denaturation at 94^0^C for 30 seconds, primer annealing at 55°C for 30 seconds, extension at 72°C for 30 seconds, and a final extension at 72°C for 10 minutes. This is shown in Table 2.

**Table 2:** Cyclic conditions used for nested PCR PCR: polymerase chain reaction

Name of Gene	Steps of PCR	Initial denaturation	Primer annealing	Extension	Final extension
SSU	1^st^ Round PCR	94°C for 5 minutes followed by 30 cycles of denaturation at 94^0^C for 45 seconds	45°C for 2 minutes	72°​​C for 1.5 minutes	72°C for 10 minutes
	2^nd^ Round PCR	94°​​​​​​​C for 5 minutes followed by 35 cycles of denaturation at 94^0^C for 30 seconds	55°​​​​​​​C for 30 seconds	72°​​​​​​​C for 30 seconds	72°​​​​​​​C for 10 minutes
gp60gene	1^st^ Round PCR	94°C for 5 minutes followed by 35 cycles of denaturation at 94°C for 30 seconds	55°C for 45 seconds	72°C for 1 minute	72°​​​​​​​C for 10 minutes
	2^nd^ Round PCR	94°​​​​​​​C for 5 minutes followed by 30 cycles of denaturation at 94°C for 30 seconds	55°​​​​​​​C for 30 seconds	72°​​​​​​​C for 30 seconds	72°​​​​​​​C for 10 minutes

*gp60 *Gene of* Cryptosporidium *Species*: *In the first round PCR,initial denaturation at 94°^ ^C for five minutes was followed by 35 cycles of denaturation at 94°​​​​​​​^ ^C for 30 seconds, primer annealing at 55°​​​​​​​C for 45 seconds, extension at 72°​​​​​​​C for one minutes, and a final extension of 72°​​​​​​​C for 10 minutes. In the second round PCR*, *initial denaturation at 94°​​​​​​​^ ^C for five minutes was followed by 30 cycles of denaturation at 94°​​​​​​​^ ^C for 30 seconds, primer annealing at 55°​​​​​​​C for 30 seconds, extension at 72°​​​​​​​ C for 30 seconds, and a final extension at 72°​​​​​​​ C for 10 minutes. This is shown in Table 2.

**Figure 4:**
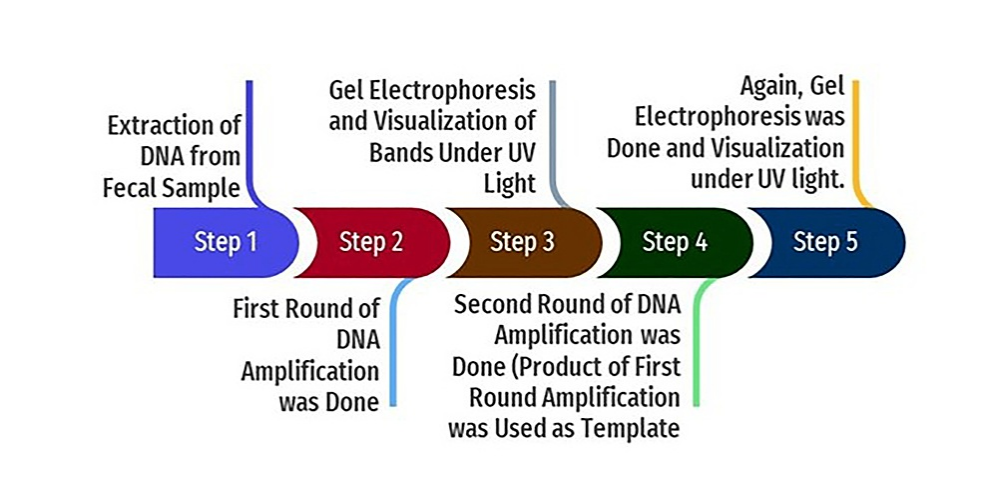
Flowchart of nested PCR PCR: polymerase chain reaction; UV: ultraviolet

In the nested PCR, the amplified product of the first round of PCR is used as the template. The second set of primers was added to the reaction mixture in the second amplification (nested PCR). The PCR reactions were conducted in a thermal cycler (Applied Biosystems, Thermo Fisher Scientific, Waltham, Massachusetts, United States). The amplicon size was determined by comparing the position of the amplicon concerning that of a 100 bp DNA ladder loaded in the adjacent well and simultaneously electrophoresis. Samples were scored as PCR positive for *Cryptosporidium* spp. when PCR product of 240 bp could be detected for *SSU rRNA* gene. Samples were detected as PCR positive for *Cryptosporidium* spp. when 350 bp could be seen for the* gp60* gene. The flowchart of nested PCR is given in Figure 4.

Data analysis

A predesigned questionnaire systematically recorded all the relevant information (history), socio-demographic history, clinical findings, and laboratory findings of every case. The data were analyzed using IBM SPSS Statistics for Windows, Version 25.0 (Released 2017; IBM Corp., Armonk, New York, United States) and Stata Statistical Software: Release 15 (2017; StataCorp LLC, College Station, Texas, United States). Statistical analysis was done by standard statistical procedure; Microsoft Excel (Microsoft Corporation, Redmond, Washington, United States) was used to prepare graphs and charts. A p-value <0.05 was considered significant. The multivariate logistic regression model was used to see the association between independent factors and *Cryptosporidium* infestation. The regression model was adjusted by age and gender.

## Results

Distribution of the study population according to their age and sex

A total of 219 fecal samples were collected from pediatric diarrheic patients at two tertiary medical college hospitals. The age range of the patients was two months to 18 years. At first, all samples were examined by direct microscopic examination, then mZN staining, and afterward, nested PCR was done for specific target genes of *Cryptosporidium*. The majority of the study population belonged to the age group of 1-5 years (47%), followed by the <1 year age group (30.1%) (Table 3). Of them, 125 (57%) were male and 94 (43%) were female. The male to female ratio was about 1.33:1.

**Table 3:** Distribution of study population by age (n=219)

Age Group	Frequency	Infected cases n(%)	Total cases (n) and percentage (%)
<1	66	2 (22.22%)	30.1%
1-5	103	7 (77.78%)	47.0%
6-10	37	0	16.9%
11-15	11	0	5.0%
15-18	02	0	0.9%
Total	219	9	100%

Microscopic assay

Wet Mount Preparation

All fecal samples were first examined for direct microscopic examination by saline and iodine preparation. Table 4 shows wet mount findings among 219 study samples where *Giardia duodenalis* was found positive in five (2.3%) samples. Moreover, other parasites like helminthic eggs were found in some samples. Ova of *Ascaris lumbricoides*, *Trichuris trichiura*, and mixed infection were detected in three (1.4%), one (0.5%), and two (0.9%), respectively.

**Table 4:** Detection of parasites by wet mount in the study population (n=219)

Parasites	Frequency	Percentage (%)
Giardia duodenalis	05	2.3
Ascaris lumbricoides	03	1.4
Trichuris trichiura	01	0.5
*Ascaris lumbricoides* and*Trichuris trichiura*	02	0.9
No parasite	208	95
Total	219	100

Identification of Cryptosporidium by mZN Staining

The microscopic examination of the stool samples through mZN staining showed the presence of *Cryptosporidium* oocysts (Figure 4) in 3/219 (1.4%) samples (Table 5).

**Figure 5:**
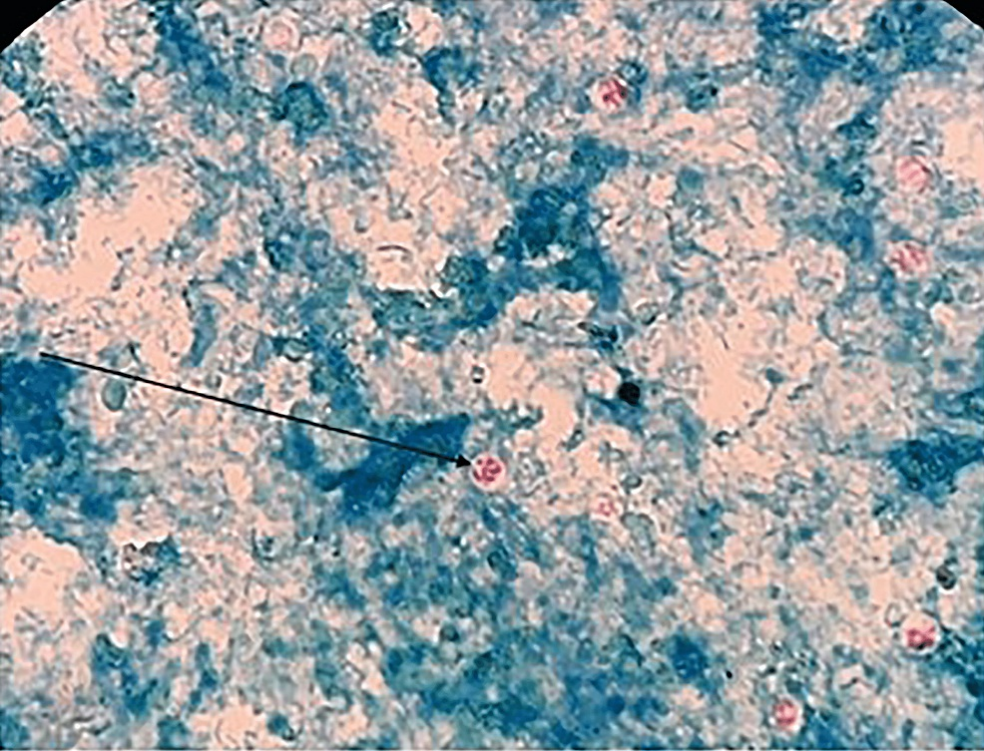
Cryptosporidium oocyst (arrow) in mZN stain mZN: modified Ziehl-Neelsen

**Table 5:** Association of microscopic findings of Cryptosporidium with nested PCR (n=219) p-value derived from chi-square test. Figures within parentheses indicate the percentage.

Microscopic findings	Nested PCR findings		Total	p value
	(+ve)	(-ve)		
Modified Ziehl-Neelsen staining				
*Cryptosporidium* (+ve)	03 (33.3%)	00 (0.0%)	03	
*Cryptosporidium* (-ve)	06 (66.7%)	210 (100%)	216	<0.001
Total	09	210	219	

Nested PCR findings

**Figure 6:**
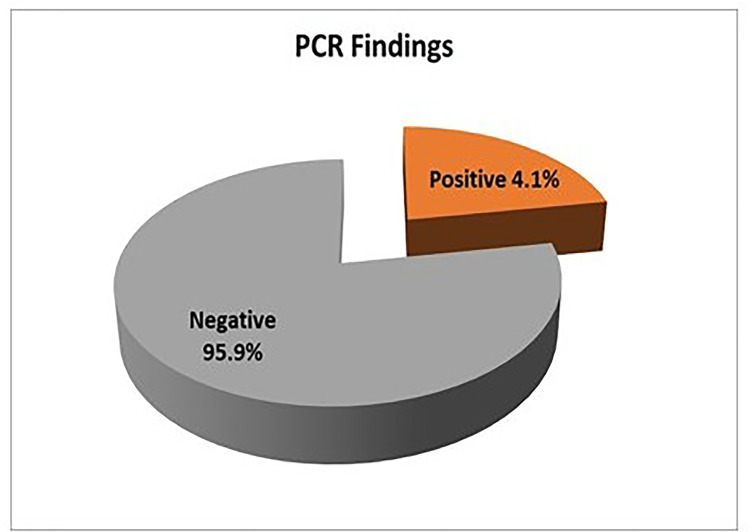
Distribution of Cryptosporidium spp. by nested PCR (n=219) PCR: polymerase chain reaction

**Figure 7:**
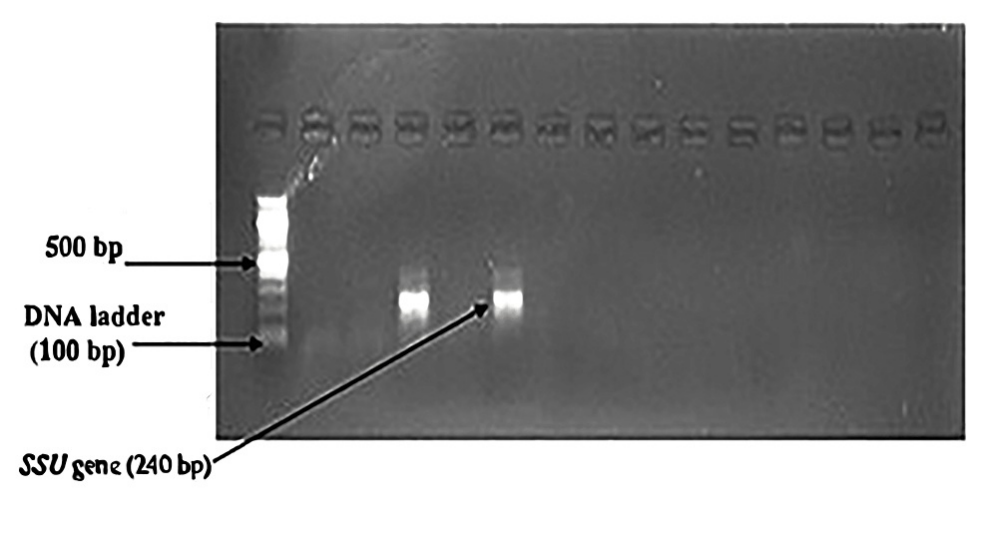
Bands of amplified DNA of Cryptosporidium spp.

**Figure 8:**
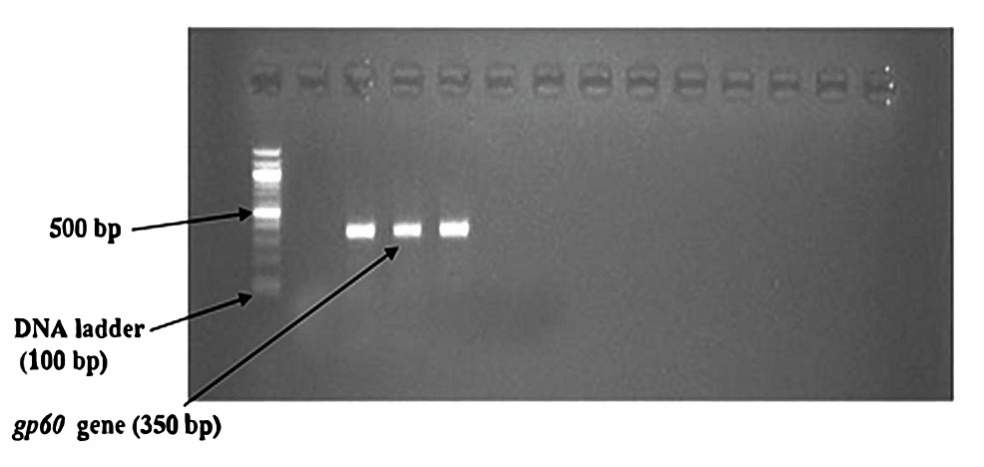
Bands of amplified DNA of Cryptosporidium spp.

Nested PCR detected *Cryptosporidium* oocysts in nine (4.1%) samples (Figure 6). The bands of *SSU-rRNA* (240 bp) and *gp60 *genes (350 bp) are shown in Figure 7 and Figure 8, respectively.

The prevalence of *Cryptosporidium* spp. was more in the 1-5 years age group, and male predominance was observed, but both were statistically non-significant. Table 4 shows the association between microscopic and nested PCR findings of* Cryptosporidium*. Here the Chi-square test was done, and it was found to be statistically significant (p<0.001). The sensitivity and specificity of *Cryptosporidium* Spp. microscopy was found to be 33.3% and 100%, respectively, taking the PCR method as the gold standard.

Multivariate logistic regression to determine the independent factors associated with *Cryptosporidium* infection

Table 6 summarizes the risk factors associated with cryptosporidiosis, including male gender, unsafe drinking water (without boiling or health risk levels of contaminants), irregular hand washing, rural residence, insufficient exclusive breastfeeding, and history of having pets. It reflected the use of unsafe drinking water had a probability of 3.91 times to be infected with *Cryptosporidium* infestation, OR= 3.91; 95% CI (1.05, 16.1); p=0.049, lack of regular hand washing had a chance of being infected with *Cryptosporidium* infestation by 9.68 times higher compared to traditional hand washing, OR= 9.68; 95% CI (1.92, 48.4) and insufficiency of exclusive breastfeeding have a risk to infected with *Cryptosporidium* infestation by 3.82 times higher compared to feed breast milk exclusively, OR=3.82(1.01, 15.3); p=0.049. But other factors like female gender (OR=1.02, P=0.972), rural residence (OR=3.46, P=0.081), and H/O having a pet (OR=0.41, P=0.237) have no significant association with the infection. Most of the study population (64.8%) drink safe water, and 70.3% use regular hand washing.

**Table 6:** Multivariate logistic regression to determine the independent factors associated with Cryptosporidium infection A multivariate logistic regression model was used to estimate the p-value. The regression model was adjusted by age and sex CI: confidence interval; OR: odds ratio; s: significant, ns: not significant

Variables	Number of samples for each category	Number of positive *Cryptosporidium* based on categories	Adjusted OR (95% CI)	P-value
Gender				
Male	125 (57.1%)	5 (55.6%)	1	
Female	94 (42.9%)	4 (44.4%)	1.02 (0.26, 3.94)	0.972
Drinking water				
Safe (boiled or appropriate filteratrate)	142 (64.8%)	3 (33.3%)	1	
Unsafe	77 (35.2%)	6 (66.7%)	3.91(1.05, 16.1)	0.049
Hand Washing				
Regular	154 (70.3)	2 (22.2%)	1	
Irregular	65 (29.7)	7 (77.8%)	9.68(1.92, 48.4)	0.006
Residence				
Urban	152 (69.4%)	4 (44.4%)	1	
Rural	67 (30.6%)	5 (55.6%)	3.46 (0.86, 13.9)	0.081
Breast feeding				
Exclusive	141 (64.4%)	3 (33.3%)	1	
Not exclusive	78 (35.6%)	6 (66.7%)	3.82 (1.01, 15.3)	0.049
H/O having pet				
No	178 (81.3%)	6 (66.7%)	1	
Yes	41 (18.7%)	3 (33.3%)	0.41 (0.10, 1.79)	0.237

## Discussion

Intestinal parasites such as *Cryptosporidium *spp. and other parasites are liable for diarrhea, especially among children in developing countries [99]. Though infections due to these parasites are self-limiting in immunocompetent individuals, chronicity often results in malnutrition, growth faltering, and cognitive function impairment, especially in children [60,100-105]. Because of these alarming effects on a child's health, it emphasizes the need to establish the incidence of protozoan parasites responsible for childhood diarrheic disease.

In this study, 2.3% cases were found *Giardia duodenalis *positive by wet mount preparation among 219 samples. The prevalence of *Giardia duodenalis* infection among 0-15 years Portuguese children was 1.9%, as estimated by direct microscopic examination. Nevertheless, when monoclonal ELISA techniques were utilized, the rate increased to 6.8% [106]. Furthermore, a higher rate (7.8%) of *Giardia duodenalis* infestation was found in the younger group (0-5 years), and there was no difference observed between the sexes [106]. A study among pediatric patients (below five years) in a tertiary hospital was done where *Giardia* cysts were found in 4.14% of cases [107]. In a slum area of Bangladesh, *Giardia duodenalis* positive samples were found in 6.01% of cases among school-going children [108]. In Sikkim, India, a prospective study among symptomatic children (<15 years) was done where *Giardia* cysts were found in 5% of cases by wet mount [109]. About 8.2% of patients were positive for giardiasis through the direct smear method in Kashmir Valley, India [110]. Another study in Lucknow, India, showed that *Giardia* was detected in 15.5% by immediate wet mount preparation [111], much higher than the current study findings. This dissimilarity may be due to the large sample size (n=1680) and large age group distribution (3 to 45 years). The geographic area may also be a factor in this difference.

In wet mount film, the current study showed that ova of *Ascaris lumbricoides, Trichuris trichiura*, and mixed infection were detected in three (1.4%), one (0.5%), and two (0.9%), respectively. A study reported 0.9% *Trichuris trichiura *[98], but no *Ascaris lumbricoides* were found in hospitalized pediatric diarrheic children (<5 years), according to our study. Another study demonstrated 0.6% of *Trichuris trichiura* ova, but *Ascaris lumbricoides* was 9% [112]. Other studies reported the prevalence of *Ascaris lumbricoides* and *Trichuris trichiura* ova as 1.5-8.2% and 0.8-0.9%, respectively [38,113]. In our research, a relatively low number of helminths was detected probably because of the urban setting of the study population, which may have had better sanitation and hygiene practices. Another cause could be the ingestion of anti-helminthic drugs at a regular interval.

Age and sex-specific vulnerability are essential for the prevalence of diarrheal illness, where various factors can contribute to this. These may include biochemical factors like hormones, enzymes, specific proteins, genetic or immunologic factors, food habits, culture, etc. [114]. *Cryptosporidium *was found positive in 1.4% of diarrheal samples by mZN staining. A surveillance study on children under 12 years old in Tehran was done where the prevalence rate of the protozoa was 1.19% [67]. Another survey on pediatric children (<5 years) reported 2.3% *Cryptosporidium* oocyst [107]. A study reflected detection rates of *Cryptosporidium *spp. from outpatients of the International Centre for Diarrhoeal Disease Research, Bangladesh (ICDDR'B) of 4.96% [41]. In Malaysia, the prevalence of these protozoa among diarrheal subjects (<12 years) was 4.62% by mZN staining [115, 116]. In Cameroon, a hospital-based cross-sectional study among children aged <5 years reflects that the prevalence of *Cryptosporidium* infection was 13.4% in microscopy (mZN staining), having the highest detection rate in the 31-60 months group [88], which is dissimilar to this study. Several factors may play a vital role in this higher prevalence of cryptosporidiosis, such as geographic region, seasonal variation, age, personal hygiene, drinking or using untreated water, no exclusive breastfeeding, low educational status, and poor socioeconomic status of the parents.

In this study, there were some limitations. There was low trophozoite and/or cyst and oocyst detection because of the empirical use of antimicrobials, especially antiprotozoal and anthelminthic. These medications are often self-medicated or given by non-graduate medical practitioners. Additionally, this study could not collect samples from the same patient on multiple occasions. Moreover, microscopic findings depend on individual expertise. Direct microscopic examination after mZN staining of Cryptosporidium relies on morphologic recognition of small-sized oocysts that may be scanty in number, which may be challenging to visualize. Again, they could be inconsistently stained, and misdiagnosis may happen. Therefore, this method is impractical to standardize as it is influenced by the microscopist's individual skills of the microscopist involved [43]. Moreover, the procedure is incapable of detecting the low parasite count. Collecting at least three stool samples on alternate days is often indicated because of intermittent shedding of protozoan cysts and oocysts. It is worth mentioning that other stages are not visible microscopy. Nevertheless, it's difficult and inconvenient to go for three samples from the same patient. Though conventional microscopy of more than one fecal sample continues to be recommended to diagnose intestinal protozoa in stool samples, its sensitivity is still low even after multiple examinations. Also, the parasite might be disguised by bile pigment and not visualized by wet mount examination [101]. Since this study was done with a single fecal sample, a low parasite concentration could be missed through microscopy. This could be the reason behind their relatively low rate of detection.

*Cryptosporidium *spp. was found positive in 4.1% of samples by nested PCR. In one study, the prevalence of *Cryptosporidium* was 4.8% among children below five years of age with diarrhea [117]. A longitudinal cohort study was done on Bangladeshi slum children where the same procedure found *Cryptosporidium *positive cases in 6.3% of cases [41]. A prospective study on children under 16 years showed that 7.7% of patients were detected by PCR [60]. Another study found the prevalence of this parasite is about 1.3% [118]. Improved sanitation, safe drinking water, and awareness about health and hygiene enable a lower prevalence of these parasitic diseases in the community [119]. Another cause may lie in examining a single fecal specimen per individual for diagnosis, which is less sensitive than using multiple samples [45,57].

Moreover, the study was performed in urban rather than rural or slum areas. PCR is considered a more sensitive and specific diagnostic method, but some limitations remain such as PCR bands were not confirmed by sequencing because of structural and financial difficulty. There is a list of PCR inhibitors in stool samples, namely lipids, hemoglobin, bilirubin, bile salts, polysaccharides from mucous, bacteria, and food degradation products, which can also affect the result of amplification. Commercial kits, including extraction columns, have been used to purify the DNA to minimize the effect of DNA inhibitors. Despite using such a kit, false-negative results may happen [120,121]. The highest cases of *Cryptosporidium* were detected in the same age group (one to five years) 7/103 (6.79%), followed by <1-year age group 2/66 (3.03%), which was not statistically significant according to the p-value (p=0.376). Moreover, we revealed from our study that all *Cryptosporidium*-positive cases were children <3 years old. In another study, it was seen that the highest prevalence of *Cryptosporidium* infection was in the age group of three to four years (14.3%), followed by <1-year age group (4.5%) [117]. A study found that *Cryptosporidium* infection was predominant among children <5 years (22%) [50]. Most infected cases were children <4 years of age [117].

Children (1-5 years) develop a habit of putting unwashed hands, toys, and other objects inside their mouths. Moreover, their compromised immunological status and poor hygiene practices are susceptible to intestinal infections, especially during this period [122,123]. The infection rate decreased in higher age groups with minimum infection rate because of the improved immunological status of the individual [40,124]. It is observed that there is a difference in detection rate with other studies. One reason could be that it was a tertiary hospital-based study rather than a rural or slum-based one. On the other side, our study's low detection rate may suggest an improved living standard for the study participants. It may be due to the improvement of the sanitation and hygiene system. The empirical use of antiparasitic drugs could be one of the reasons behind the low prevalence of intestinal parasites in our country [125,126]. Failure to detect intestinal protozoan parasites may be due to seasonal variation and the intermittent nature of excretion of this parasite in the stool [48,127].

In this study, male predominance was observed in the case of *Cryptosporidium *Spp. Infection was similar to Hawash et al. [128], but the opposite picture was found in another study where females were affected more [129,130]. This discrimination is unclear because under-five children of both sexes are engaged in the same recreational activity and are likely exposed to the same environmental conditions. Still, it could be because the males constitute most of the study population. The association of microscopic findings with PCR for pathogens showed high statistical significance (p<0.001). The sensitivity of *Cryptosporidium* microscopy was 33.3%, and specificity was 100% in the present study. Two other studies found similar validity [131,132].

Globally, foodborne cryptosporidiosis has been recognized as the main reason for such infestation [133-135]. Additionally, drinking water from swimming pools, waterparks, fountains, lakes, and rivers contaminated with *Cryptosporidium *remains one of the common causes of spreading cryptosporidiosis [136-138]. This water source could be contaminated by fecal matter. In some city areas, sewage and toilet wastage may get into the surface water, which could be a potential concern for the fecal-oral transmission of this protozoan parasite. Furthermore, cryptosporidiosis has been positioned fifth among the 24 most significant foodborne parasites [139,140]. The World Bank reported that 98% of Bangladeshi people currently have access to better-quality water. However, water quality remains poor across the country because 80% possess microbial contamination [141]. United Nations Children's Fund (UNICEF)/WHO reported that about 25% of Bangladeshi people do not have safe drinking water resources in their homes [142]. The results of risk factor analysis support the role of having unsafe drinking water in *Cryptosporidium *infection that is statistically significant (p-value=0.043), which agrees with another study conducted in Pakistan [143]. Another critical issue is that *Cryptosporidium *oocysts are highly resistant to numerous disinfectant agents [143-145] and are killed by boiling water above 70° C in less than one minute [146]. In addition, multiple studies, including the WHO guidelines for drinking water quality, suggest that those oocysts become non-viable and inactivated at 60-71.7° C [129,135,143,147,148].

Another salient factor behind protozoal diarrhea is inadequate hygiene practices like handwashing [62,63,149-151]. Our study found a significant association between poor hand washing practice and cryptosporidiosis (p-value=0.001). It may be due to improper washing of hands while handling infant feeding bottles. Moreover, there is a tradition of practicing force-feeding techniques with bare hands to ensure enough food intake by the children to support their growth [152,153]. The hazard of bare hands (not properly washed) enables direct transmission of this foodborne protozoan disease [154,155]. These malpractices encourage ingesting food and water contaminated with oocysts and are shed from infected individuals [156,157].

Undoubtedly, breastfeeding has a widespread impact on children's health conditions, and there remains a significant correlation between breastfeeding and diarrhea [158-160]. Our study speculated that failure of exclusive breastfeeding was associated with *Cryptosporidium* infection [130,161]. It can be postulated that the mother’s milk provides numerous antibodies, Immunoglobulin A (IgA), which offers passive protective immunity against various parasitic infections [130,161,162]. Multiple studies reported that parasitic-infested infection is almost prevented till six months of age and when complementary foods are associated with infective diarrhea [117,163,164]. Though contact with animals has been reported as a risk factor for this infection [165-167], we observed a higher distribution of this pathogen in children without animal rearing. Our findings were similar to earlier studies [44,168]. The finding suggests the transmission mode could be the person-to-person contact-no association of the pathogen with the rural resident, supporting some previous studies [118,124,169,170].

## Conclusions

The present research can be brought into play that the PCR approach demonstrated better results than the traditional microscopic procedure as it yields more pragmatic outcomes. This procedure also denotes that it can adequately diagnose the parasitic causes of diarrhea among children. Consequently, it brings considerable advantages to the treatment of pediatric diarrheal patients in Bangladesh and other low and middle-income countries (LMICs). As a result, irrational prescribing and self-medication of antimicrobials hopefully be minimized. Thereby, it is expected that the rate of antimicrobial resistance will be cut down. Moreover, this research provides scientific data to detect other protozoal parasitic diarrhea through mentioned methods. Additionally, this study enables information regarding the prevalence of protozoan causes of diarrhea in the locality or region of Bangladesh.

The samples were identified positively by mZN staining for *Cryptosporidium* spp.; oocyst was successfully proved positive by molecular assay (PCR), which recommends the practice of PCR approaches and conventional microscopic procedures to overthrow the practice the diagnostic drawbacks. So, the staining method should be combined with wet mount preparation as a routine examination for stool to detect and diagnose the parasite effectively. Moreover, molecular analysis is also recommended where facilities are available. This would help to diagnose the protozoa precisely. Further molecular analysis like DNA sequencing can be done, which will provide us with specific information about the species and genotypes, hence, lending the researcher a transparent idea about the epidemiology to identify risk factors, mode of transmission, pathogenicity, and genetic diversity, etc.

Parasites are one of the critical causes of pediatric diarrheic disease in the Bangladeshi community. mZN staining should be introduced as a routine-based detection method for *Cryptosporidium *spp. mZN is much more cost-effective in considering those countries that have poor budgetary allocation and access to healthcare. Finally, the practice of regular hand washing and consumption of boiled water is an essential factor in minimizing parasitic infection, including cryptosporidiosis

## References

[1] Sweetser S (2012). Evaluating the patient with diarrhea: a case-based approach. Mayo Clin Proc.

[2] Corinaldesi R, Stanghellini V, Barbara G, Tomassetti P, De Giorgio R (2012). Clinical approach to diarrhea. Intern Emerg Med.

[3] Nemeth V, Pfleghaar N (2022). Diarrhea. StatPearls [Internet].

[4] Siciliano V, Nista EC, Rosà T, Brigida M, Franceschi F (2020). Clinical management of infectious diarrhea. Rev Recent Clin Trials.

[5] DuPont HL (2016). Persistent diarrhea: a clinical review. JAMA.

[6] Hegde S, Benoit SR, Arvelo W (2019). Burden of laboratory-confirmed shigellosis infections in Guatemala 2007-2012: results from a population-based surveillance system. BMC Public Health.

[7] Rabbani GH, Ahmed S, Hossain I, Islam R, Marni F, Akhtar M, Majid N (2009). Green banana reduces clinical severity of childhood shigellosis: a double-blind, randomized, controlled clinical trial. Pediatr Infect Dis J.

[8] Varre JV (2021). Vaccines are not one size fits all, just like medications: rotavirus vaccine study. Clin Exp Vaccine Res.

[9] Gould CV, File TM Jr, McDonald LC (2015). Causes, burden, and prevention of Clostridium difficile Infection. Infect Dis Clin Pract (Baltim Md).

[10] Liu J, Platts-Mills JA, Juma J (2016). Use of quantitative molecular diagnostic methods to identify causes of diarrhoea in children: a reanalysis of the GEMS case-control study. Lancet.

[11] Soares-Weiser K, Maclehose H, Ben-Aharon I, Goldberg E, Pitan F, Cunliffe N (2010). Vaccines for preventing rotavirus diarrhoea: vaccines in use. Cochrane Database Syst Rev.

[12] (2022). World Health Organization: Diarrhoeal disease. https://www.who.int/news-room/fact-sheets/detail/diarrhoeal-disease.

[13] Ugboko HU, Nwinyi OC, Oranusi SU, Oyewale JO (2020). Childhood diarrhoeal diseases in developing countries. Heliyon.

[14] Boschi-Pinto C, Velebit L, Shibuya K (2008). Estimating child mortality due to diarrhoea in developing countries. Bull World Health Organ.

[15] (2022). World Health Organization: Children: improving survival and well-being. https://www.who.int/news-room/fact-sheets/detail/children-reducing-mortality.

[16] Demissie GD, Yeshaw Y, Aleminew W, Akalu Y (2021). Diarrhea and associated factors among under five children in sub-Saharan Africa: evidence from demographic and health surveys of 34 sub-Saharan countries. PLoS One.

[17] Pinkerton R, Oriá RB, Lima AA (2016). Early childhood diarrhea predicts cognitive delays in later childhood independently of malnutrition. Am J Trop Med Hyg.

[18] Khalil IA, Troeger C, Rao PC (2018). Morbidity, mortality, and long-term consequences associated with diarrhoea from Cryptosporidium infection in children younger than 5 years: a meta-analyses study. Lancet Glob Health.

[19] Troeger C, Colombara DV, Rao PC (2018). Global disability-adjusted life-year estimates of long-term health burden and undernutrition attributable to diarrhoeal diseases in children younger than 5 years. Lancet Glob Health.

[20] Fagundes-Neto U (2013). Persistent diarrhea: still a serious public health problem in developing countries. Curr Gastroenterol Rep.

[21] Melese B, Paulos W, Astawesegn FH, Gelgelu TB (2019). Prevalence of diarrheal diseases and associated factors among under-five children in Dale District, Sidama zone, Southern Ethiopia: a cross-sectional study. BMC Public Health.

[22] Mokomane M, Kasvosve I, de Melo E, Pernica JM, Goldfarb DM (2018). The global problem of childhood diarrhoeal diseases: emerging strategies in prevention and management. Ther Adv Infect Dis.

[23] Kotloff KL (2017). The burden and etiology of diarrheal illness in developing countries. Pediatr Clin North Am.

[24] Chakravarty I, Bhattacharya A, Das SK (2017). Water, sanitation and hygiene: the unfinished agenda in the World Health Organization South-East Asia Region. WHO South East Asia J Public Health.

[25] Squire SA, Ryan U (2017). Cryptosporidium and Giardia in Africa: current and future challenges. Parasit Vectors.

[26] Kundu S, Kundu S, Banna MH, Ahinkorah BO, Seidu AA, Okyere J (2022). Prevalence of and factors associated with childhood diarrhoeal disease and acute respiratory infection in Bangladesh: an analysis of a nationwide cross-sectional survey. BMJ Open.

[27] Hasan MZ, Mehdi GG, De Broucker G (2021). The economic burden of diarrhea in children under 5 years in Bangladesh. Int J Infect Dis.

[28] Ahmed S, Dorin F, Satter SM (2021). The economic burden of rotavirus hospitalization among children < 5 years of age in selected hospitals in Bangladesh. Vaccine.

[29] Nguyen TV, Le Van P, Le Huy C, Weintraub A (2004). Diarrhea caused by rotavirus in children less than 5 years of age in Hanoi, Vietnam. J Clin Microbiol.

[30] LeClair CE, McConnell KA (2022). Rotavirus. StatPearls [Internet].

[31] Tian L, Zhu X, Chen Z (2016). Characteristics of bacterial pathogens associated with acute diarrhea in children under 5 years of age: a hospital-based cross-sectional study. BMC Infect Dis.

[32] Zhou Y, Zhu X, Hou H (2018). Characteristics of diarrheagenic Escherichia coli among children under 5 years of age with acute diarrhea: a hospital based study. BMC Infect Dis.

[33] Adam MA, Wang J, Enan KA (2018). Molecular survey of viral and bacterial causes of childhood diarrhea in Khartoum State, Sudan. Front Microbiol.

[34] Zhu XH, Tian L, Cheng ZJ (2016). Viral and bacterial etiology of acute diarrhea among children under 5 years of age in Wuhan, China. Chin Med J (Engl).

[35] Wang X, Wang J, Sun H (2015). Etiology of childhood infectious diarrhea in a developed region of China: compared to childhood diarrhea in a developing region and adult diarrhea in a developed region. PLoS One.

[36] Boughattas S, Behnke JM, Al-Ansari K, Sharma A, Abu-Alainin W, Al-Thani A, Abu-Madi MA (2017). Molecular analysis of the enteric protozoa associated with acute diarrhea in hospitalized children. Front Cell Infect Microbiol.

[37] Osman M, El Safadi D, Cian A (2016). Prevalence and risk factors for intestinal protozoan infections with Cryptosporidium, Giardia, Blastocystis and Dientamoeba among schoolchildren in Tripoli, Lebanon. PLoS Negl Trop Dis.

[38] Davlin SL, Jones AH, Tahmina S (2020). Soil-transmitted helminthiasis in four districts in Bangladesh: household cluster surveys of prevalence and intervention status. BMC Public Health.

[39] Ryan U, Zahedi A, Paparini A (2016). Cryptosporidium in humans and animals-a one health approach to prophylaxis. Parasite Immunol.

[40] Bouzid M, Hunter PR, Chalmers RM, Tyler KM (2013). Cryptosporidium pathogenicity and virulence. Clin Microbiol Rev.

[41] Khan WA, Rogers KA, Karim MM (2004). Cryptosporidiosis among Bangladeshi children with diarrhea: a prospective, matched, case-control study of clinical features, epidemiology and systemic antibody responses. Am J Trop Med Hyg.

[42] Shirley DA, Moonah SN, Kotloff KL (2012). Burden of disease from cryptosporidiosis. Curr Opin Infect Dis.

[43] Baptista RP, Cooper GW, Kissinger JC (2021). Challenges for cryptosporidium population studies. Genes (Basel).

[44] Krumkamp R, Aldrich C, Maiga-Ascofare O (2021). Transmission of Cryptosporidium species among human and animal local contact networks in sub-saharan Africa: a multicountry study. Clin Infect Dis.

[45] O'Leary JK, Sleator RD, Lucey B (2021). Cryptosporidium spp. diagnosis and research in the 21st century. Food Waterborne Parasitol.

[46] Firoozi Z, Sazmand A, Zahedi A (2019). Prevalence and genotyping identification of Cryptosporidium in adult ruminants in central Iran. Parasit Vectors.

[47] Dabas A, Shah D, Bhatnagar S, Lodha R (2017). Epidemiology of Cryptosporidium in pediatric diarrheal illnesses. Indian Pediatr.

[48] Leitch GJ, He Q (2012). Cryptosporidiosis-an overview. J Biomed Res.

[49] Di Genova BM, Tonelli RR (2016). Infection strategies of intestinal parasite pathogens and host cell responses. Front Microbiol.

[50] Semrad CE (2012). Approach to the patient with diarrhea and malabsorption. Goldman's Cecil Medicine.

[51] (2022). Clinical Methods: The History, Physical, and Laboratory Examinations, 3rd Edition. https://www.ncbi.nlm.nih.gov/books/NBK414/.

[52] Hodges K, Gill R (2010). Infectious diarrhea: cellular and molecular mechanisms. Gut Microbes.

[53] Thiagarajah JR, Donowitz M, Verkman AS (2015). Secretory diarrhoea: mechanisms and emerging therapies. Nat Rev Gastroenterol Hepatol.

[54] Kirkpatrick BD, Noel F, Rouzier PD (2006). Childhood cryptosporidiosis is associated with a persistent systemic inflammatory response. Clin Infect Dis.

[55] Kiela PR, Ghishan FK (2016). Physiology of intestinal absorption and secretion. Best Pract Res Clin Gastroenterol.

[56] Checkley W, White AC Jr, Jaganath D (2015). A review of the global burden, novel diagnostics, therapeutics, and vaccine targets for cryptosporidium. Lancet Infect Dis.

[57] Khurana S, Chaudhary P (2018). Laboratory diagnosis of cryptosporidiosis. Trop Parasitol.

[58] Collinet-Adler S, Ward HD (2010). Cryptosporidiosis: environmental, therapeutic, and preventive challenges. Eur J Clin Microbiol Infect Dis.

[59] Sharma P, Khurana S, Sharma A, Sehgal R, Malla N (2016). Presence of intracellular viruses in human Cryptosporidium isolates. Ann Parasitol.

[60] Desai NT, Sarkar R, Kang G (2012). Cryptosporidiosis: an under-recognized public health problem. Trop Parasitol.

[61] Moore CE, Elwin K, Phot N (2016). Molecular characterization of Cryptosporidium species and Giardia duodenalis from symptomatic Cambodian children. PLoS Negl Trop Dis.

[62] Luby SP, Rahman M, Arnold BF (2018). Effects of water quality, sanitation, handwashing, and nutritional interventions on diarrhoea and child growth in rural Bangladesh: a cluster randomised controlled trial. Lancet Glob Health.

[63] Ercumen A, Mertens A, Arnold BF (2018). A cluster-randomized controlled trial, the effects of water, sanitation, handwashing, and nutritional interventions on child enteric protozoan infections in rural Bangladesh. Environ Sci Technol.

[64] Sow SO, Muhsen K, Nasrin D (2016). The burden of cryptosporidium diarrheal disease among children < 24 months of age in moderate/high mortality regions of Sub-Saharan Africa and South Asia, utilizing data from the global enteric multicenter study (GEMS). PLoS Negl Trop Dis.

[65] Hussain G, Roychoudhury S, Singha B, Paul J (2017). Incidence of Cryptosporidium andersoni in diarrheal patients from southern Assam, India: a molecular approach. Eur J Clin Microbiol Infect Dis.

[66] Areeshi MY, Beeching NJ, Hart CA (2007). Cryptosporidiosis in Saudi Arabia and neighboring countries. Ann Saudi Med.

[67] Khurana S, Sharma P, Sharma A, Malla N (2012). Evaluation of Ziehl-Neelsen staining, auramine phenol staining, antigen detection enzyme linked immunosorbent assay and polymerase chain reaction, for the diagnosis of intestinal cryptosporidiosis. Trop Parasitol.

[68] Kalantari N, Ghaffari S, Bayani M (2018). Cryptosporidium spp. infection in Iranian children and immunosuppressive patients: a systematic review and meta-analysis. Caspian J Intern Med.

[69] Casemore DP, Armstrong M, Sands RL (1985). Laboratory diagnosis of cryptosporidiosis. J Clin Pathol.

[70] Chalmers RM, Atchison C, Barlow K, Young Y, Roche A, Manuel R (2015). An audit of the laboratory diagnosis of cryptosporidiosis in England and Wales. J Med Microbiol.

[71] Rusnak J, Hadfield TL, Rhodes MM, Gaines JK (1989). Detection of Cryptosporidium oocysts in human fecal specimens by an indirect immunofluorescence assay with monoclonal antibodies. J Clin Microbiol.

[72] Ghoshal U, Jain V, Dey A, Ranjan P (2018). Evaluation of enzyme linked immunosorbent assay for stool antigen detection for the diagnosis of cryptosporidiosis among HIV negative immunocompromised patients in a tertiary care hospital of northern India. J Infect Public Health.

[73] Chalmers RM, Campbell BM, Crouch N, Charlett A, Davies AP (2011). Comparison of diagnostic sensitivity and specificity of seven Cryptosporidium assays used in the UK. J Med Microbiol.

[74] Robinson G, Chalmers RM (2020). Cryptosporidium diagnostic assays: microscopy. Methods Mol Biol.

[75] Morgan UM, Pallant L, Dwyer BW, Forbes DA, Rich G, Thompson RC (1998). Comparison of PCR and microscopy for detection of Cryptosporidium parvum in human fecal specimens: clinical trial. J Clin Microbiol.

[76] Cunha FS, Peralta RH, Peralta JM (2019). New insights into the detection and molecular characterization of Cryptosporidium with emphasis in Brazilian studies: a review. Rev Inst Med Trop Sao Paulo.

[77] Xiao L (2009). Overview of Cryptosporidium presentations at the 10th International Workshops on Opportunistic Protists. Eukaryot Cell.

[78] Khan A, Shaik JS, Grigg ME (2018). Genomics and molecular epidemiology of Cryptosporidium species. Acta Trop.

[79] Mohammad SM, Ali MS, Abdel-Rahman SA, Moustafa RA, Sarhan MH (2021). Genotyping of Cryptosporidium species in children suffering from diarrhea in Sharkyia Governorate, Egypt. J Infect Dev Ctries.

[80] Jex AR, Smith HV, Monis PT, Campbell BE, Gasser RB (2008). Cryptosporidium--biotechnological advances in the detection, diagnosis and analysis of genetic variation. Biotechnol Adv.

[81] Jex AR, Gasser RB (2009). Diagnostic and analytical mutation scanning of Cryptosporidium: utility and advantages. Expert Rev Mol Diagn.

[82] Gilchrist CA, Cotton JA, Burkey C (2018). Genetic diversity of Cryptosporidium hominis in a Bangladeshi community as revealed by whole-genome sequencing. J Infect Dis.

[83] Steiner KL, Ahmed S, Gilchrist CA (2018). Species of Cryptosporidia causing subclinical infection associated with growth faltering in rural and urban Bangladesh: a birth cohort study. Clin Infect Dis.

[84] Hira KG, Mackay MR, Hempstead AD (2011). Genetic diversity of Cryptosporidium spp. from Bangladeshi children. J Clin Microbiol.

[85] Ahmed T, Khanum H, Uddin MS, Barua P, Arju T, Kabir M, Haque R (2016). Entamoeba histolytica, Giardia lamblia, and Cryptosporidium Spp. Infection in children in an urban slum area of Bangladesh. Biores Commun.

[86] Moore SR, Lima NL, Soares AM (2010). Prolonged episodes of acute diarrhea reduce growth and increase risk of persistent diarrhea in children. Gastroenterology.

[87] Billah SM, Raihana S, Ali NB (2019). Bangladesh: a success case in combating childhood diarrhoea. J Glob Health.

[88] Tombang AN, Ambe NF, Bobga TP (2019). Prevalence and risk factors associated with cryptosporidiosis among children within the ages 0-5 years attending the Limbe regional hospital, southwest region, Cameroon. BMC Public Health.

[89] (2022). Centers for Disease Control and Prevention (CDC): Stool specimens - extraction of parasite DNA from fecal specimens using FastDNA® kit. https://www.cdc.gov/dpdx/diagnosticprocedures/stool/dnaextraction.html.

[90] Becker SL, Lohourignon LK, Speich B (2011). Comparison of the Flotac-400 dual technique and the formalin-ether concentration technique for diagnosis of human intestinal protozoon infection. J Clin Microbiol.

[91] Jember TH, Amor A, Nibret E (2022). Prevalence of Strongyloides stercoralis infection and associated clinical symptoms among schoolchildren living in different altitudes of Amhara National Regional State, northwest Ethiopia. PLoS Negl Trop Dis.

[92] Cheesbrough M (2022). Parasitological tests. District Laboratory Practice in Tropical Countries. Part 1, Second Edition.

[93] Rahman HU, Khan W, Mehmood SA (2021). Prevalence of cestodes infection among school children of urban parts of Lower Dir district, Pakistan. Braz J Biol.

[94] Selek MB, Bektöre B, Karagöz E, Baylan O, Özyurt M (2016). Distribution of parasites detected in stool samples of patients admitted to our parasitology laboratory during a three-year period between 2012 and 2014. Turkiye Parazitol Derg.

[95] Casemore DP (1991). ACP Broadsheet 128: June 1991. Laboratory methods for diagnosing cryptosporidiosis. J Clin Pathol.

[96] Yanta CA, Bessonov K, Robinson G, Troell K, Guy RA (2021). CryptoGenotyper: a new bioinformatics tool for rapid Cryptosporidium identification. Food Waterborne Parasitol.

[97] Ikenaga M, Katsuragi S, Handa Y, Katsumata H, Chishaki N, Kawauchi T, Sakai M (2018). Improvements in bacterial primers to enhance selective SSU rRNA gene amplification of plant-associated bacteria by applying the LNA oligonucleotide-PCR clamping technique. Microbes Environ.

[98] Koehler AV, Haydon SR, Jex AR, Gasser RB (2016). Cryptosporidium and Giardia taxa in faecal samples from animals in catchments supplying the city of Melbourne with drinking water (2011 to 2015). Parasit Vectors.

[99] Bauhofer AF, Cossa-Moiane I, Marques S (2020). Intestinal protozoan infections among children 0-168 months with diarrhea in Mozambique: June 2014 - January 2018. PLoS Negl Trop Dis.

[100] Coutinho BP, Oriá RB, Vieira CM (2008). Cryptosporidium infection causes undernutrition and, conversely, weanling undernutrition intensifies infection. J Parasitol.

[101] Costa LB, JohnBull EA, Reeves JT (2011). Cryptosporidium-malnutrition interactions: mucosal disruption, cytokines, and TLR signaling in a weaned murine model. J Parasitol.

[102] Guerrant DI, Moore SR, Lima AA, Patrick PD, Schorling JB, Guerrant RL (1999). Association of early childhood diarrhea and cryptosporidiosis with impaired physical fitness and cognitive function four-seven years later in a poor urban community in northeast Brazil. Am J Trop Med Hyg.

[103] Aldeyarbi HM, Abu El-Ezz NM, Karanis P (2016). Cryptosporidium and cryptosporidiosis: the African perspective. Environ Sci Pollut Res Int.

[104] Mahmoudi MR, Ongerth JE, Karanis P (2017). Cryptosporidium and cryptosporidiosis: the Asian perspective. Int J Hyg Environ Health.

[105] Rafferty ER, Schurer JM, Arndt MB, Choy RK, de Hostos EL, Shoultz D, Farag M (2017). Pediatric cryptosporidiosis: an evaluation of health care and societal costs in Peru, Bangladesh and Kenya. PLoS One.

[106] Kialashaki E, Fakhar M, Sharif M, Daryani A, Saberi R (2020). The mixed AII and BIII genotypes of human Giardia lamblia isolate circulating in Mazandaran Province, Northern Iran. Infect Disord Drug Targets.

[107] Pervin MK, Jhora ST, Paul S, Naher A, Sarkar D (2019). Causative agents for diarrhea in under 5 children in a tertiary care hospital. Bang Med J Khulna.

[108] Hossain MR, Musa S, Zaman RF, Khanum H (2019). Occurrence of intestinal parasites among school-going children of a slum area in Dhaka city. Bang J Zool.

[109] Chanu NO, Singh TS, Dutta S (2018). Detection and genetic characterization of Giardia intestinalis in children with gastrointestinal symptoms by PCR RFLP in Sikkim, India. J Nat Sc Biol Med.

[110] Abdullah I, Tak H, Ahmad F, Gul N (2016). Prevalence and associated risk factors for Giardiasis among children in district Anantnag of Kashmir Valley, India. J Gastro Hepato Dis.

[111] Jahan N, Khatoon R, Ahmad S (2014). A comparison of microscopy and enzyme linked immunosorbent assay for diagnosis of Giardia lamblia in human faecal specimens. J Clin Diagn Res.

[112] Rahman M, Islam M, Doza S (2022). Higher helminth ova counts and incomplete decomposition in sand-enveloped latrine pits in a coastal sub-district of Bangladesh. PLoS Negl Trop Dis.

[113] Adu-Gyasi D, Asante KP, Frempong MT (2018). Epidemiology of soil transmitted Helminth infections in the middle-belt of Ghana, Africa. Parasite Epidemiol Control.

[114] Alam S, Khanum H, Zaman RF, Haque R (2019). : Prevalence of different protozoan parasites in patients visiting at ICDDR’B Hospital, Dhaka. J Asiat Soc Bangladesh, Sci.

[115] Rossle NF, Latif B, Malik AS, Fadzli FM, Abu NA (2012). Cryptosporidiosis among children with diarrhea admitted to Hospital Selayang and Hospital, Sungai Buloh, Selangor, Malaysia. J Trop Med Parasitol.

[116] Latif B, Rossle NF (2015). Cryptosporidiosis among children with diarrhea in three Asian countries: a review. Asian Pac J Trop Biomed.

[117] Anejo-Okopi JA, Okojokwu JO, Ebonyi AO (2016). Molecular characterization of cryptosporidium in children aged 0- 5 years with diarrhea in Jos, Nigeria. Pan Afr Med J.

[118] Ysea MA, Umaña MC, Fuentes SP, Campos IV, Carmona MC (2022). Standardization of molecular techniques for the detection and characterization of intestinal protozoa and other pathogens in humans. J Venom Anim Toxins Incl Trop Dis.

[119] Njambi E, Magu D, Masaku J, Okoyo C, Njenga SM (2020). Prevalence of intestinal parasitic infections and associated water, sanitation, and hygiene risk factors among school children in Mwea Irrigation Scheme, Kirinyaga County, Kenya. J Trop Med.

[120] Anuar TS, Azreen SN, Salleh FM, Moktar N (2014). Molecular epidemiology of giardiasis among Orang Asli in Malaysia: application of the triosephosphate isomerase gene. BMC Infect Dis.

[121] Ahmed W, Simpson SL, Bertsch PM (2022). Minimizing errors in RT-PCR detection and quantification of SARS-CoV-2 RNA for wastewater surveillance. Sci Total Environ.

[122] Bourke CD, Berkley JA, Prendergast AJ (2016). Immune dysfunction as a cause and consequence of malnutrition. Trends Immunol.

[123] Bloomfield SF, Stanwell-Smith R, Crevel RW, Pickup J (2006). Too clean, or not too clean: the hygiene hypothesis and home hygiene. Clin Exp Allergy.

[124] Putignani L, Menichella D (2010). Global distribution, public health and clinical impact of the protozoan pathogen cryptosporidium. Interdiscip Perspect Infect Dis.

[125] Ashigbie PG, Shepherd S, Steiner KL (2021). Use-case scenarios for an anti-Cryptosporidium therapeutic. PLoS Negl Trop Dis.

[126] Miyamoto Y, Eckmann L (2015). Drug development against the major diarrhea-causing parasites of the small intestine, Cryptosporidium and Giardia. Front Microbiol.

[127] Costa D, Razakandrainibe R, Basmaciyan L (2022). A summary of cryptosporidiosis outbreaks reported in France and overseas departments, 2017-2020. Food Waterborne Parasitol.

[128] Hawash Y, Dorgham LSh, Al-Hazmi AS, Al-Ghamdi MS (2014). Prevalence of Cryptosporidium-associated diarrhea in a high altitude-community of Saudi Arabia detected by conventional and molecular methods. Korean J Parasitol.

[129] Hunter PR, Nichols G (2002). Epidemiology and clinical features of Cryptosporidium infection in immunocompromised patients. Clin Microbiol Rev.

[130] Pedersen SH, Wilkinson AL, Andreasen A (2014). Cryptosporidium prevalence and risk factors among mothers and infants 0 to 6 months in rural and semi-rural Northwest Tanzania: a prospective cohort study. PLoS Negl Trop Dis.

[131] Uppal B, Singh O, Chadha S, Jha AK (2014). A comparison of nested PCR assay with conventional techniques for diagnosis of intestinal cryptosporidiosis in AIDS cases from northern India. J Parasitol Res.

[132] Kaushik K, Khurana S, Wanchu A, Malla N (2008). Evaluation of staining techniques, antigen detection and nested PCR for the diagnosis of cryptosporidiosis in HIV seropositive and seronegative patients. Acta Trop.

[133] Rossle NF, Latif B (2013). Cryptosporidiosis as threatening health problem: a review. Asian Pac J Trop Biomed.

[134] Ursini T, Moro L, Requena-Méndez A, Bertoli G, Buonfrate D (2020). A review of outbreaks of cryptosporidiosis due to unpasteurized milk. Infection.

[135] Innes EA, Chalmers RM, Wells B, Pawlowic MC (2020). A one health approach to tackle Cryptosporidiosis. Trends Parasitol.

[136] Pignata C, Bonetta S, Bonetta S, Cacciò SM, Sannella AR, Gilli G, Carraro E (2019). Cryptosporidium oocyst contamination in drinking water: a case study in Italy. Int J Environ Res Public Health.

[137] Chalmers RM (2012). Waterborne outbreaks of cryptosporidiosis. Ann Ist Super Sanita.

[138] Xiao S, An W, Chen Z, Zhang D, Yu J, Yang M (2012). The burden of drinking water-associated cryptosporidiosis in China: the large contribution of the immunodeficient population identified by quantitative microbial risk assessment. Water Res.

[139] (2022). Food and Agriculture Organization of the United Nations and the World Health Organization. Multicriteria-based ranking for risk management of food-borne parasites. WHO Press, World Health Organization, 20 Avenue App, 1211 Geneva 27, Switzerland,. Multicriteria-based ranking for risk management of food-borne parasites.

[140] Ryan U, Hijjawi N, Xiao L (2018). Foodborne cryptosporidiosis. Int J Parasitol.

[141] (2022). Bangladesh: Access to clean water will reduce poverty faster. https://www.worldbank.org/en/news/press-release/2018/10/11/bangladesh-access-to-clean-water-will-reduce-poverty-faster.

[142] UNICEF/WHO Report. 61.7 million deprived of basic hygiene facilities in Bangladesh (2022). 61.7 Million Deprived of Basic Hygiene Facilities in Bangladesh: UN report. UN.

[143] Khan A, Shams S, Khan S, Khan MI, Khan S, Ali A (2019). Evaluation of prevalence and risk factors associated with Cryptosporidium infection in rural population of district Buner, Pakistan. PLoS One.

[144] Quilez J, Sanchez-Acedo C, Avendaño C, del Cacho E, Lopez-Bernad F (2005). Efficacy of two peroxygen-based disinfectants for inactivation of Cryptosporidium parvum oocysts. Appl Environ Microbiol.

[145] Chauret CP, Radziminski CZ, Lepuil M, Creason R, Andrews RC (2001). Chlorine dioxide inactivation of Cryptosporidium parvum oocysts and bacterial spore indicators. Appl Environ Microbiol.

[146] Naciri M, Mancassola R, Fort G, Danneels B, Verhaeghe J (2011). Efficacy of amine-based disinfectant KENO™COX on the infectivity of Cryptosporidium parvum oocysts. Vet Parasitol.

[147] (2015). World Health Organization. Boil Water. Technical brief. WHO/FWC/WSH/15.02. (20150115202022). Technical Brief: Boil Water.

[148] Fayer R (2004). Cryptosporidium: a water-borne zoonotic parasite. Vet Parasitol.

[149] Berhe B, Bugssa G, Bayisa S, Alemu M (2018). Foodborne intestinal protozoan infection and associated factors among patients with watery diarrhea in Northern Ethiopia; a cross-sectional study. J Health Popul Nutr.

[150] Lin A, Arnold BF, Mertens AN (2017). Effects of water, sanitation, handwashing, and nutritional interventions on telomere length among children in a cluster-randomized controlled trial in rural Bangladesh. Elife.

[151] Knee J, Sumner T, Adriano Z (2018). Risk factors for childhood enteric infection in urban Maputo, Mozambique: A cross-sectional study. PLoS Negl Trop Dis.

[152] Guldan GS, Zeitlin MF, Beiser AS, Super CM, Gershoff SN, Datta S (1993). Maternal education and child feeding practices in rural Bangladesh. Soc Sci Med.

[153] Moore AC, Akhter S, Aboud FE (2006). Responsive complementary feeding in rural Bangladesh. Soc Sci Med.

[154] Todd EC (2014). Foodborne diseases: overview of biological hazards and foodborne diseases. Encyclopedia of Food Safety.

[155] Schirone M, Visciano P, Tofalo R, Suzzi G (2016). Editorial: biological hazards in food. Front Microbiol.

[156] Aniesona AT, Bamaiyi PH (2014). Retrospective study of cryptosporidiosis among diarrhoeic children in the arid region of north-eastern Nigeria. Zoonoses Public Health.

[157] Ryan U, Fayer R, Xiao L (2014). Cryptosporidium species in humans and animals: current understanding and research needs. Parasitology.

[158] Delelegn MW, Endalamaw A, Belay GM (2020). Determinants of acute diarrhea among children under-five in Northeast Ethiopia: unmatched case-control study. Pediatric Health Med Ther.

[159] Siregar AY, Pitriyan P, Walters D (2018). The annual cost of not breastfeeding in Indonesia: the economic burden of treating diarrhea and respiratory disease among children (< 24mo) due to not breastfeeding according to recommendation. Int Breastfeed J.

[160] Turin CG, Ochoa TJ (2014). The role of maternal breast milk in preventing infantile diarrhea in the developing world. Curr Trop Med Rep.

[161] Korpe PS, Liu Y, Siddique A (2013). Breast milk parasite-specific antibodies and protection from amebiasis and cryptosporidiosis in Bangladeshi infants: a prospective cohort study. Clin Infect Dis.

[162] Abdel-Hafeez EH, Belal US, Abdellatif MZ, Naoi K, Norose K (2013). Breast-feeding protects infantile diarrhea caused by intestinal protozoan infections. Korean J Parasitol.

[163] Palmieri JR, Meacham SL, Warehime J (2018). Relationships between the weaning period and the introduction of complementary foods in the transmission of gastrointestinal parasitic infections in children in Honduras. Res Rep Trop Med.

[164] Fawzy A, Arpadi S, Kankasa C (2011). Early weaning increases diarrhea morbidity and mortality among uninfected children born to HIV-infected mothers in Zambia. J Infect Dis.

[165] Pumipuntu N, Piratae S (2018). Cryptosporidiosis: a zoonotic disease concern. Vet World.

[166] Olabanji GM, Maikai BV, Otolorin GR (2016). Prevalence and risk factors associated with faecal shedding of Cryptosporidium oocysts in dogs in the Federal Capital Territory, Abuja, Nigeria. Vet Med Int.

[167] Chukwu VE, Daniels OO, Olorunfemi JC, Opara MN (2019). Cryptosporidium oocysts: prevalence in dogs in Abuja, Federal Capital Territory, Nigeria. Ann Parasitol.

[168] Chuah CJ, Ziegler AD (2018). Temporal variability of faecal contamination from on-site sanitation systems in the groundwater of northern Thailand. Environ Manage.

[169] Zueter A (2020). The status of cryptosporidiosis in Jordan: a review. East Mediterr Health J.

[170] Robertson LJ, Johansen ØH, Kifleyohannes T, Efunshile AM, Terefe G (2020). Cryptosporidium infections in Africa-how important is zoonotic transmission? A review of the evidence. Front Vet Sci.

